# Evaluating the Age-Dependent Potential for Protein Deposition in Naked Neck Meat Type Chicken

**DOI:** 10.3390/ani5010056

**Published:** 2015-01-20

**Authors:** Daulat R. Khan, Christian Wecke, Ahmad R. Sharifi, Frank Liebert

**Affiliations:** 1 Division Animal Nutrition Physiology, Department of Animal Sciences, Georg-August-University, Kellnerweg 6, 37077 Goettingen, Germany; E-Mails: dkhan@gwdg.de (D.R.K.); cwecke@gwdg.de (C.W.); 2 Division Animal Breeding and Genetics, Department of Animal Sciences, Georg-August-University, Albrecht-Thaer-Weg 3, 37075 Goettingen, Germany; E-Mail: rsharif@gwdg.de

**Keywords:** naked neck meat type chicken, N utilization model, N maintenance requirement, protein deposition potential

## Abstract

**Simple Summary:**

Growth rates of fast-growing chickens are reduced by a higher ambient temperature (AT) because of difficulties in dissipating heat through the feather coverage. Naked neck meat type genotypes could be helpful in increasing the tolerance for high AT. However, basic model parameters of this genotype necessary to further assess amino acid requirements are as yet unavailable. The experiments were conducted to estimate both the daily nitrogen maintenance requirement (NMR) and the potential for daily nitrogen retention NR_max_T). These observed model parameters provide the basic information to characterize the growth potential of the genotype for further application in modeling of individual amino acid requirements of naked neck meat type chicken.

**Abstract:**

The introduction of the naked neck gene (*Na*) into modern meat type chicken is known to be helpful in increasing the tolerance for a high ambient temperature (AT) by reducing the feather coverage which allows for a higher level of heat dissipation compared to normally feathered (*na*/*na*) birds. In addition, reduced feather coverage could affect requirements for sulfur containing amino acids. As a prerequisite for further modeling of individual amino acid requirements, the daily N maintenance requirement (NMR) and the threshold value of daily N retention (NR_max_T) were determined. This was carried out using graded dietary protein supply and exponential modeling between N intake (NI) and N excretion (NEX) or N deposition (ND), respectively. Studies with homozygous (*Na*/*Na*) and heterozygous (*Na*/*na*) naked neck meat type chicken utilized 144 birds of average weight (50% of each genotype and sex) within two N balance experiments during both the starter (days 10–20) and the grower period (days 25–35). Birds were randomly allotted to five diets with graded dietary protein supply but constant protein quality. The observed estimates depending on genotype, sex and age varied for NMR and NR_max_T from 224 to 395 and 2881 to 4049 mg N/BW_kg_^0.67^/day, respectively.

## 1. Introduction

In the last few decades, substantial genetic progress has been made in broiler growth, feed efficiency, meat yield and increased metabolic rate [1,2]. Conversely, the birds became more sensitive to suboptimal environmental conditions, especially to hot ambient condition [3]. Compared with muscle growth, the cardiovascular and respiratory systems do not adapt as well to extreme environment conditions, which resulted in a lower capability to regulate energy expenditure and the body water balance system [2]. There are two different main strategies for improving the tolerance of chicken for higher ambient temperatures (AT). Firstly, by reducing temperatures through convection, conduction, radiation and by maintaining house temperature using different management practices, or by decreasing heat production [4]. However, some factors may lead to higher production costs [5]. Secondly, through the introduction of breeds well adapted to high AT and also showing a higher growth potential. Such a breeding and selection strategy can be particularly effective to achieve improved results in unfavorable tropical environments. A useful breeding strategy is to create a breeding stock carrying a major gene for better heat tolerance. The naked neck dominant gene (*Na*) is known to improve heat endurance through different pathways: Either by decreasing the metabolic heat increment or by diminishing the insulating power of bird’s plumage which leads to higher heat loss and thus ultimately helps in regulating body temperature [4]. Previous studies investigated the crude protein (CP) requirement for naked neck birds, but furnished only uncertain information. According to the high protein content of feathers, Ajang *et al.* [6] suggested that the degree of feathering in fast and slow feathering broilers may influence the CP requirement of chicken. Yalcin *et al.* [7] observed that naked neck birds did not require less dietary protein because of their reduced feather covering. However, conclusive amino acid (AA) requirement studies have not been carried out for the naked neck birds.

Further applications of an exponential modeling procedure [8,9,10,11,12,13] were utilized to fill this gap. This study requires experimental data regarding both the age dependent potential for nitrogen deposition and the daily nitrogen maintenance requirement of this particular genotype. It was the aim of the study to predict these model parameters for homozygous and heterozygous (*Na*/*Na*; *Na*/*na*) naked neck meat type chicken depending on age and sex.

## 2. Experimental Section

### 2.1. Stock and Husbandry

The experiments were conducted at the Division of Animal Nutrition Physiology, Department of Animal Sciences at the Georg-August-University Goettingen with the approval of the Animal Welfare Law Committee of Lower Saxony, Germany.

Heterozygous parent stock (*Na*/*na*) from a heavy broiler sire line (Aviagen^®^ Poultry Breeders UK) with a genetic disposition for high growth performance was used for obtaining desired experimental homozygous (*Na*/*Na*); and heterozygous (*Na*/*na*) chicks. The only *Na/Na* and *Na*/*na* genotypes used in the present studies were the full sib and half sib offspring of heterozygous (*Na*/*na*) naked neck parents. These share the same genetic background to a high degree, which facilitated the accurate measurement of N deposition for both the genetics during starter and grower period.

All of the experimental birds were reared under standardized housing and feeding regimes from day 0 to 5 and 0 to 20. Afterwards, a total of 144 birds of average weight (72 *Na*/*Na* and 72 *Na*/*na*, each 50% male and female) were selected for N balance experiments involving both starter (days 10–20; 2 × 36 birds) and grower period (days 25–35; 2 × 36 birds). The birds were individually housed in metabolic cages with wire floors, individual feeders and self-drinking systems. The room temperature was gradually reduced from 32 °C to 23 °C with increasing age. Humidity was maintained between 60%–70% and monochromatic light was provided for 23 h following 1 h darkness.

### 2.2. Feeding and Sampling

Chickens of both genotypes and sexes were randomly allotted to five experimental diets (N1–N5). The formulation of the pelleted diets was based on a constant mixture of the native feed protein sources of soy protein concentrate (SPC), wheat, maize and wheat gluten, but diluted with wheat starch to create graded crude protein (CP) levels between 10% and 38% (Table 1 and Table 2). For future applications of the data, methionine (Met) was set as first limiting amino acid (LAA) to calculate requirement data following principles of the diet dilution technique. Except Met, other amino acid (AA) requirements were fulfilled according to NRC [14] recommendations and the achieved dietary amino acid pattern was kept unchanged independently of dietary protein levels at both the age periods (Table 2). According to age dependent energy demands, the energy content of the grower diets was enhanced and the CP contents were slightly decreased without changing the AA profile. The dietary Ca and P supply was 1.0% and 0.6% in starter and 0.85% and 0.55% in grower feed, respectively. Further mineral and vitamin supplementations were in line with current recommendations [14,15].

Two experimental periods, *i.e.*, starter (days 10–20) and grower period (days 25–35) were conducted for every genotype and sex. The individual experimental period was divided into an adaptation period (5 days) and two consecutive quantitative excreta collecting periods (each 5 days). At the beginning of the adaptation period, feed was given *ad libitum* to estimate the adequate level of individual feed intake under housing conditions in the metabolic cages. The individual feed supply was kept constant from day 3 of the adaptation period, slightly adapted during the first two days of the collecting period, and kept constant again up to the end of the collecting periods, respectively. To avoid effects of feed rejection by individual birds due to the extreme CP contents, N balance studies were conducted with 10 and 8 replicates for the lowest (N1) and highest CP diets (N5) and 6 replicates (diets N2–4) per genotype, sex and age period, respectively. Excreta collection was conducted twice a day to prevent ammonia loss from non-acidified excreta. Excreta samples were immediately frozen (−20 °C) for further analysis.

**Table 1:** Composition of experimental diets (percentage as fed).

Ingredients	Diets									
	Starter (Days 10–20)					Grower (Days 25–35)				
Diet ^1^	N1	N2	N3	N4	N5	N1	N2	N3	N4	N5
Maize	6.62	10.59	14.56	18.45	22.50	5.95	9.70	13.67	18.08	22.05
Wheat	5.09	8.14	11.19	14.19	17.30	4.58	7.46	10.51	13.90	16.95
Soy protein concentrate	10.15	16.23	22.32	28.29	34.50	9.13	14.88	20.96	27.72	33.81
Fish meal	1.91	3.06	4.21	5.33	6.50	1.72	2.80	3.95	5.22	6.37
Wheat gluten	1.76	2.82	3.88	4.92	6.00	1.59	2.59	3.65	4.82	5.88
Soybean oil	3.09	4.94	6.79	8.61	10.50	3.24	5.28	7.44	9.84	12.00
Cellulose	1.80	1.35	0.90	0.46	-	1.82	1.40	0.95	0.45	-
Wheat starch	65.54	49.17	32.78	16.72	-	68.42	52.70	35.99	17.45	0.78
Premix ^2^	1.00	1.00	1.00	1.00	1.00	1.00	1.00	1.00	1.00	1.00
DCP	2.50	2.08	1.65	1.25	0.80	2.30	1.89	1.45	0.97	0.55
CaCO_3_	0.30	0.40	0.52	0.60	0.74	0.06	0.15	0.28	0.41	0.50
NaCl	0.24	0.22	0.20	0.18	0.16	0.19	0.15	0.15	0.12	0.10

^1^ Starter = N1: 10.85; N2: 17.44; N3: 24.10; N4:30.70; N5:37.63 % CP on DM basis; grower = N1: 9.75; N2: 15.96; N3: 22.59; N4:30.05; N5:36.83percent crude protein on dry matter basis. ^2^ Provided (per kilogram of diet): Vitamin A, 12,000 IU; vitamin D_3_, 3,500 IU; vitamin E, 40 mg; thiamin, 2.5 mg; riboflavin, 8.0 mg; vitamin B_6_, 6.0 mg; vitamin B_12_, 32 μg; vitamin K_3_, 4.5 mg; nicotinic acid, 45 mg; CaCO_3_, 15 mg; folic acid, 1.2 mg; biotin, 50 μg; choline chloride, 550 mg; Mn, 100 mg; Zn, 80 mg; Fe, 30 mg; Cu, 20 mg; I, 1.2 mg; Co, 0.4 mg; Se, 0.4 mg; and butylated hydroxytoluene, 100 mg.

**Table 2:** Analyzed nutrient content and amino acid composition of experimental diets (percent on dry matter basis).

Nutrients	Diets									
	Starter (Days 10–20)					Grower (Days 25–35)				
Diet	N1	N2	N3	N4	N5	N1	N2	N3	N4	N5
Crude protein	10.85	17.44	24.10	30.70	37.63	9.75	15.96	22.59	30.05	36.83
Ether extract	3.94	6.26	8.62	10.96	13.41	4.04	6.55	9.24	12.25	14.99
Crude fiber	2.35	2.36	2.37	2.39	2.40	2.30	2.31	2.33	2.34	2.35
Crude ash	5.43	5.86	6.32	6.75	7.24	4.78	5.14	5.62	6.11	6.55
N-free extract	70.69	60.59	50.13	40.87	30.40	72.48	62.95	52.67	41.18	30.75
Starch	65.02	55.65	46.17	36.78	26.89	70.35	60.04	49.75	37.58	27.16
Total sugars	1.64	1.64	1.64	1.63	1.63	1.74	1.70	1.69	1.64	1.61
AMEn^1^ (MJ/kg)	14.73	15.5	15.33	15.16	15.37	15.02	15.21	15.43	15.47	15.59
**Amino Acids**	**Amino Acid Composition (g/100g Crude Protein)**					**Amino Acid Ratio (Lys = 100)**				
Lys	5.09					100				
Met	1.44					28				
Cys	1.46					29				
Thr	3.63					71				
Trp	0.96					19				
Arg	6.26					123				
Ile	4.03					79				
Leu	7.50					147				
Val	4.24					83				
Phe	4.71					92				

^1^ Nitrogen corrected apparent metabolizable energy, calculated according to WPSA [16].

### 2.3. Laboratory Analysis

Dietary ingredients, experimental diets and excreta were analyzed according to the German standards [17]. The N content was quantified using to the Dumas method (Leco^®^ LP-2000, Leco^®^ Instrument GmbH, Kirchheim, Germany) and CP was calculated with factor 6.25. Amino acids (AA) of the protein sources were analyzed by ion-exchange chromatography (Biochrom^®^ 30, Biochrom Ltd. Cambridge, UK) following acid hydrolysis with and without an oxidation step for quantitative determination of sulfur-containing amino acids. Ether extract was analyzed following HCl hydrolysis of the feed samples.

### 2.4. Model Parameter Assessment and Statistics

Model applications basically require actual information regarding the daily N maintenance requirement (NMR) and the threshold value for daily N retention (NR_max_T). The NMR estimation utilized the exponential regression between N intake (NI) and daily N excretion (NEX) following graded dietary protein supply [8,9,10,11,18,19,20] in order to prevent non-physiological N free feeding conditions. The obtained NMR value was set as point of intersection with the y-axis. Accordingly, estimating of threshold value NR_max_T utilized the regression of N deposition (ND) depending on NI:

NR = NR_max_T (1 − e^−b·NI^) (1)

ND = NR_max_T (1 − e^−b·NI^) − NMR (2)

where NR = daily N retention (ND + NMR; mg/BW_kg_^0.67^); NR_max_T = theoretical maximum for daily NR (mg/BW_kg_^0.67^); ND = daily N deposition (mg/BW_kg_^0.67^); NMR = daily nitrogen maintenance requirement (mg/BW_kg_^0.67^); NI = daily N intake (mg/BW_kg_^0.67^); e = basic number of natural logarithm (ln); *b* = slope of the N retention curve (parameter of feed protein quality).

The model parameter *b* as an indicator of the dietary protein quality was derived following logarithmization and transformation of Equation (1):

*b* = [ln NR_max_T − ln (NR_max_T − NR)]/NI (3)

where *b* = model parameter for dietary protein quality; ln = natural logarithm; NR_max_T = theoretical maximum for daily NR; NR = nitrogen retention; NI = nitrogen intake.

Further details of this model application for evaluating the dietary protein quality independent on varying NI are reported elsewhere [13,19,21].

ND_max_T as the theoretical maximum for N deposition (ND_max_T + NMR = NR_max_T) was estimated following several iteration steps by the Levenberg-Marquardt algorithm within the SPSS statistical package. The attribute “theoretical” (T) indicates that the estimated threshold value is not in the area of practical growth data, but they characterize the estimated genetic potential that is not attainable due to several limitations in dietary and environmental factors. An overview about the applied modeling approach is given by Liebert [21].

Statistical analyses were run with SPSS. N balance data were analyzed by GLM ANOVA using the GLM procedure of SPSS software package (version 19.0 for Windows, IBM SPSS Statistics, Inc., Chicago, IL, USA). A multifactorial ANOVA was utilized using the main factors diet, genotypes, sex and age period. However, the only two-way interaction between selected variables revealed significant effects therefore the three and four way interactions were excluded. The comparison of means was carried out using LS-Means statements and adjusted by Tukey’s test. Statistical analysis was performed according to the following model including main factors and significant interaction:

y_ijklm_ = μ + D_i_ + G_j_ + S_k_+ A_l_ + DG_ij_ + DS_ik_ + DA_il_+ GS_jk_ + GA_jl_+ SA_kl_ + e_ijklm_ (4)

where y_ijklm_ is the dependent variable; μ is the general mean; D_i_ is the main effect of diet (i = N1 … N5); G_j_ is the main effect of genotype (*Na*/*Na vs. Na*/*na*); S_k_ is the main effect of sex (male *vs.* female); A_l_ is the main effect of age (starter *vs.* grower); DG, DS, DA, GA, GS and SA are the two-way interaction between the main effects and e_ijklm_ is the random error.

## 3. Results and Discussion

Results of the analysis of variance involving all diets, genotypes, sexes and age periods and their two-way interactions are given in Table 3. There was no significant main effect of genotypes. The genotypes’ interactions with age period and sex revealed non-significant differences for all N balance parameters, mean BW and DM intake. However, interactions of genotype with the experimental diets (D) showed significant differences in NEX for different diets across genotype. Sex as a main effect did not indicate any significant effects for BW, DMI and NI. However, NEX and ND were significantly different between male and female chickens. Significant interaction of sex with the diet resulted in a significant difference for NI and ND. Interaction of sex with age periods revealed significant differences for MBW, DMI, NI and NEX except ND.

The average data from N balance studies depending on diet, genotype, sex and age period are summarized in Table 4 and Table 5. MBW and DMI in starter period (d 10–20) were significantly (*p* < 0.001) lower in the diet with lowest CP level (N1) than in diets N2–N5 with increased CP levels in male and female chickens of both genotypes (Table 4). According to earlier studies [8,9,11,13,20], NI and NEX increased significantly with increasing CP level in the experimental diets. ND for diet N1 was significantly lower compared to diets with higher CP contents. Diets N3–N5 yielded non-significant differences for ND among them.

**Table 3:** Results derived from multi-factorial analysis of variance for all genotypes, sexes and age periods.

	Diet	Geno-Type	Sex	Age Period	Diet × Genotype	Diet × Sex	Diet × Age	Genotype × Sex	Genotype × Age	Sex × Age
	Probabilities									
BW (g)	<0.001	0.633	0.274	<0.001	0.956	0.945	0.002	0.432	0.547	<0.001
DMI (g/d)	<0.001	0.827	0.559	<0.001	0.985	0.882	0.001	0.327	0.578	<0.001
NI ^1^	<0.001	0.844	0.055	<0.001	0.579	0.001	<0.001	0.635	0.952	0.002
NEX ^1^	<0.001	0.113	<0.001	<0.001	0.002	0.120	<0.001	0.103	0.384	0.004
ND ^1^	<0.001	0.079	<0.001	<0.001	0.552	0.007	<0.001	0.030	0.366	0.194
*b*-value ^2^	<0.001	<0.001	<0.001	<0.001	0.328	0.950	0.017	0.183	0.914	0.001

BW = Body weight, DMI = Dry matter intake, NI = N intake, NEX = N excretion, ND = N deposition (NI–NEX). ^1^ mg/BW_kg_^0.67^ per day; ^2^* b*-value = Model parameter indicating protein quality (*b*·10^6^).

**Table 4:** Results of N balance experiments depending on dietary protein content, genotype and sex during starter period (days 10–20) ^1^.

Item	Diet (Crude Protein as % of Dry Matter)				
	N1 (10.8)	N2 (17.4)	N3 (24.1)	N4 (30.7)	N5 (37.6)
	**Males (*Na*/*Na*)**				
BW (g)	152^a^ ± 7	312^b^ ± 40	381^b^ ± 58	375^b^ ± 74	389^b^ ± 46
DMI (g/d)	17.9^a^ ± 1.1	47.1^b^ ± 4.7	57.1^b^ ± 7.1	49.9^b^ ± 7.8	51.4^b^ ± 4.7
NI ^2^	1110^a^ ± 72	2895^b^ ± 115	4213^c^ ± 96	4770^d^ ± 171	5856^e^ ± 109
NEX ^2^	412^a^ ± 33	1023^b^ ± 59	1781^c^ ± 58	2168^d^ ± 66	3187^e^ ± 44
ND ^2^	697^a^ ± 53	1872^b^ ± 99	2432^c^ ± 43	2602^c^ ± 183	2669^c^ ± 114
*b*-value ^3^	267^a^ ± 7	291^a^ ±12	299^a^ ± 4	311^a^ ± 36	274^a^ ± 29
	**Females (*Na*/*Na*)**				
BW (g)	246^a^ ± 14	425^b^ ± 52	555^b^ ±72	544^b^ ± 65	478^b^ ± 58
DMI (g/d)	31.2^a^ ± 2.1	64.6^b^ ± 4.5	68.2^b^ ±5.3	65.7^b^ ± 4.6	56.7^b^ ± 4.6
NI ^2^	1388^a^ ± 77	3246^b^ ± 68	3942^c^ ±67	4917^d^ ± 86	5659^e^ ± 85
NEX ^2^	589^a^ ± 30	1348^b^ ± 51	1752^c^ ±63	2402^d^ ± 68	3084^e^ ± 108
ND ^2^	799^a^ ± 54	1898^b^ ± 47	2190^bc^ ±36	2515^c^ ± 35	2575^c^ ±105
*b*-value ^3^	256^a^ ± 5	270^a^ ± 7	273^a^ ± 7	276^a^ ± 5	258^a^ ± 24
	**Males (*Na*/*na*)**				
BW (g)	185^a^ ± 8	321^ab^ ± 40	410^b^ ± 58	388^b^ ± 63	430^b^± 58
DMI (g/d)	23.2^a^ ± 1.2	49.3^b^ ± 4.6	58.1^b^ ± 7.1	54.0^b^ ± 7.1	53.6^b^ ± 5.4
NI ^2^	1249^a^ ± 54	2970^b^ ± 109	4066^c^ ± 111	5020^d^ ± 134	5718^e^ ±95
NEX ^2^	388^a^ ± 26	891^b^ ± 40	1609^c^ ± 62	2368^d^ ± 140	2787^e^ ± 109
ND^2^	862^a^ ± 44	2079^b^ ± 93	2458^c^ ± 56	2652^cd^ ± 51	2931^d^ ±80
*b*-value ^3^	257^a^ ± 5	294^a^ ± 9	278^a^ ± 4	259^a^ ± 11	282^a^ ± 17
	**Females (*Na*/*na*)**				
BW (g)	241^a^ ± 18	409^ab^ ± 48	494^b^ ± 63	521^b^ ±62	484^b^ ± 52
DMI (g/d)	33.0^a^ ± 3.0	61.4^b^ ± 4.2	65.1^b^ ± 5.0	61.0^b^ ± 3.7	56.4^b^ ± 3.7
NI ^2^	1483^a^ ± 98	3162^b^ ± 85	4076^c^ ± 60	4721^d^ ± 151	5605^e^ ±98
NEX ^2^	666^a^ ± 31	1300^b^ ± 64	1867^c^ ± 54	2405^d^ ± 119	2956^e^ ± 95
ND ^2^	817^a^ ± 70	1862^b^ ± 70	2209^c^ ± 61	2316^c^ ± 129	2648^d^ ± 45
*b*-value ^3^	241^a^ ± 3	258^a^ ± 9	253^a^ ± 9	238^a^ ± 17	249^a^ ± 8

BW = Body weight, DMI = Dry matter intake, NI = N intake, NEX = N excretion, ND = N deposition (NI–NEX); ^1^ LS-Means ± standard error of means (SEM) according to one-way ANOVA ^2^ mg/BW_kg_^0.67^ per day; ^3^* b*-value: Model parameter indicating the dietary protein quality (*b*·10^6^); ^d^ LS-Mean values with different superscripts within rows are significantly different (*p* < 0.05)

The MBW of naked neck chickens during grower period (days 25–35) depending on diet, genotype and sex was significantly higher in the groups with increased dietary CP levels (Table 5). However, the DMI of homozygous female and heterozygous male chickens was statistically non-significant despite differences in diet. NI and NEX increased significantly from N1–N5 in both the genotypes and sexes. Similarly, ND was increased with elevated dietary CP contents. Significantly improved ND values were generally found between diets N1 to N3. As observed in the starter period, the *b*-values of all experimental diets were insignificantly different (*p* > 0.05) independent on varying dietary CP and NI levels for both of genotypes and sexes.

**Table 5:** Results of N balance experiments depending on dietary protein content, genotype and sex during grower period (days 25–35) ^1^.

Item	Diet (Crude Protein as % of Dry Matter)				
	N1 (9.8)	N2 (16.0)	N3 (22.6)	N4 (30.0)	N5 (36.8)
	**Males (*Na*/*Na*)**				
BW (g)	1187^a^ ± 45	1519^abc^ ± 93	1526^ac^ ± 90	1722^c^ ± 114	1604^c^ ± 137
DMI (g/d)	98.2^a^ ± 6.1	133.2^b^ ± 7.0	112.8^a^ ± 3.4	116.4^a^ ± 5.4	109.7^a^ ± 6.7
NI^2^	1362^a^ ± 67	2571^b^ ± 41	3091^c^ ± 105	3899^d^ ± 57	4718^e^ ± 100
NEX ^2^	608^a^ ± 43	1162^b^ ± 25	1404^c^ ± 59	1844^d^ ± 77	2606^e^ ± 48
ND ^2^	754^a^ ± 31	1409^b^ ± 40	1688^c^ ± 72	2055^d^ ± 41	2111^d^ ± 69
*b*-value ^3^	288^a^ ± 8	282^a^ ± 6	295^a^ ± 10	315^b^ ± 12	273^a^ ± 10
	**Females (*Na*/*Na*)**				
BW (g)	1180^a^ ± 44	1346^ab^ ± 68	1511^bc^ ± 88	1544^bc^ ± 103	1741^c^ ± 79
DMI (g/d)	99.5^a^ ± 3.5	108.1^a^ ± 5.2	112.9^a^ ± 2.8	103.9^a^ ± 3.0	107.3^a^ ± 1.8
NI ^2^	1392^a^ ± 39	2265^b^ ± 83	3109^c^ ± 52	3763^d^ ± 116	4380^e^ ± 112
NEX ^2^	694^a^ ± 22	1083^b^ ± 83	1498^c^ ± 60	1990^d^ ± 70	2565^e^ ± 132
ND ^2^	698^a^ ± 22	1182^b^ ± 31	1611^c^ ± 45	1773^c^ ± 81	1816^c^ ± 64
*b*-value ^3^	339^a^ ± 5	349^a^ ± 15	382^a^ ± 16	374^a^ ± 22	335^a^ ± 23
	**Males (*Na*/*na*)**				
BW (g)	1223^a^ ± 44	1418^ab^ ± 83	1577^b^ ± 96	1576^b^ ± 166	1704^b^ ± 93
DMI (g/d)	101.0^a^ ± 5.2	122.3^a^ ± 6.6	121.3^a^ ± 4.4	111.7^a^ ± 8.6	111.0^a^± 3.6
NI ^2^	1381^a^ ± 65	2471^b^ ± 64	3245^c^ ± 67	3967^d^ ± 92	4593^e^± 53
NEX ^2^	591^a^ ± 17	1158^b^ ± 45	1431^c^ ± 61	1867^d^ ± 51	2439^e^± 54
ND ^2^	790^a^ ± 64	1313^b^ ± 38	1814^c^ ± 35	2100^c^ ± 45	2154^c^± 58
*b*-value ^3^	283^a^ ± 7	259^a^ ± 6	295^a^ ± 8	301^a^ ± 5	273^a^± 11
	**Females (*Na*/*na*)**				
BW (g)	1138^a^ ± 48	1368^ab^ ± 68	1513^b^ ± 104	1536^b^ ± 99	1594^b^ ± 74
DMI (g/d)	88.1^a^ ± 7.4	116.4^bc^ ± 3.7	112.7^c^ ± 3.1	105.0^abc^ ± 2.5	100.4^abc^ ± 2.5
NI^2^	1255^a^ ± 90	2414^b^ ± 34	3105^c^ ± 65	3814^d^ ± 149	4346^e^ ± 82
NEX^2^	651^a^ ± 26	1187^b^ ± 40	1491^c^ ± 43	1999^d^ ± 82	2490^e^ ± 86
ND^2^	603^a^ ± 68	1227^b^ ± 46	1613^c^ ± 37	1815^c^ ± 83	1855^c^ ± 43
*b*-value^3^	319^a^ ± 5	318^a^ ± 12	350^a^ ± 8	346^a^ ± 16	314^a^ ± 12

BW = Body weight, DMI = Dry matter intake, NI = N intake, NEX = N excretion, ND = N deposition (NI–NEX). ^1^ LS-Means ± standard error of means (SEM) according to one-way ANOVA. ^2^ mg/BW_kg_^0.67^ per day. ^3^* b*-value: Model parameter indicating the dietary protein quality (*b* 10^6^). ^a–d^ LS^_^Mean values with different superscripts within rows are significantly different (*p* < 0.05).

As expected, the *b*-values (model parameter *b* multiplied by 10^6^) calculated according to Equation (3) based on experimental observed ND and derived NMR and NR_max_T data for corresponding genotypes and sexes (Table 6), were not significantly different between graded NI levels, respectively. This current observation gives further support for applications of the model parameter *b* as an excellent measure of the dietary protein quality [20,21] under conditions of not sufficiently standardized NI.

**Table 6:** Model parameters for fast growing naked neck chicken related to age period, genotype and sex.

	Starter period (days 10–20)				Grower period (days 25–35)			
	*Na*/*Na*		*Na*/*na*		*Na*/*Na*		*Na*/*na*	
	Males	Females	Males	Females	Males	Females	Males	Females
NMR ^1^	262	348	224	392	341	384	346	395
NR_max_T ^2^	3763	3857	3965	4049	3397	2881	3512	3034
ND_max_T ^3^	3501	3509	3741	3657	3056	2497	3166	2639
PD_max_T ^4^	13.8	13.8	14.7	14.4	25.1	20.5	26.0	21.6
	**Probabilities**							
	NMR ^1^		NR_max_T ^2^		ND_max_T ^3^		PD_max_T ^4^	
Genotype	0.912		0.626		0.663		0.827	
Sex	0.035		0.545		0.419		0.572	
Age	0.192		0.005		0.004		<0.001	

^1^ NMR = daily N maintenance requirement (mg/BW_kg_^0.67^). ^2^ NR_max_T = theoretical maximum of daily N retention (mg/BW_kg_^0.67^). ^3^ ND_max_T = theoretical maximum of daily N deposition (mg/BW_kg_^0.67^). ^4^ PD_max_T = theoretical maximum of protein deposition (g/d) for chicken at 500g BW (starter period) and 1.500g BW (grower period).

### 3.1. Nitrogen Maintenance Requirement (NMR)

**Figure 1:**
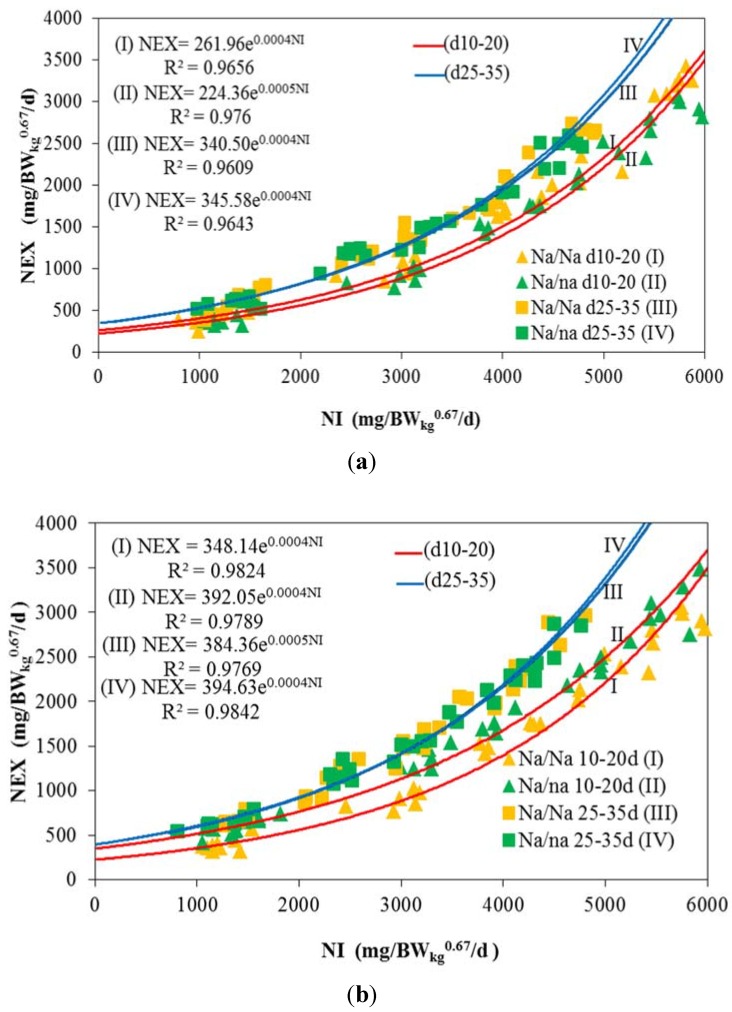
Estimation of N maintenance (NMR) by fitting exponential function between daily N intake (NI) and daily N excretion (NEX) following graded protein supply depending on sex, genotype and age period. (**a**) Males; (**b**) Females.

Results of the exponential fitting of NEX against NI provide a mathematical approach of the NEX at N free feeding level (Figure 1). The observed mean daily NMR for male (293 mg/BW_kg_^0.67^) and female chickens (380 mg/BW_kg_^0.67^) of genotypes under study are summarized in Table 6. These threshold values are higher than in previous studies with normally feathered counterparts (GRRS [15]: 264 mg/BW_kg_^0.67^; Samadi and Liebert [8]: 252 mg/BW_kg_^0.67^; Pastor *et al*. [11]: Starter 113, grower 215 mg/BW_kg_^0.67^).

In addition, GRRS [15] observed a high variability of NMR data in the literature (172 to 591 mg/BW_kg_^0.67^), depending on genotype, environment and applied design. The statistical analysis yielded a significant effect of sex on NMR. Genotype and age period were identified as non-significant factors of influence (Table 6). Observed NMR data were used for further calculation of NR_max_T.

### 3.2. Nitrogen Deposition Potential (ND_max_T)

**Figure 2:**
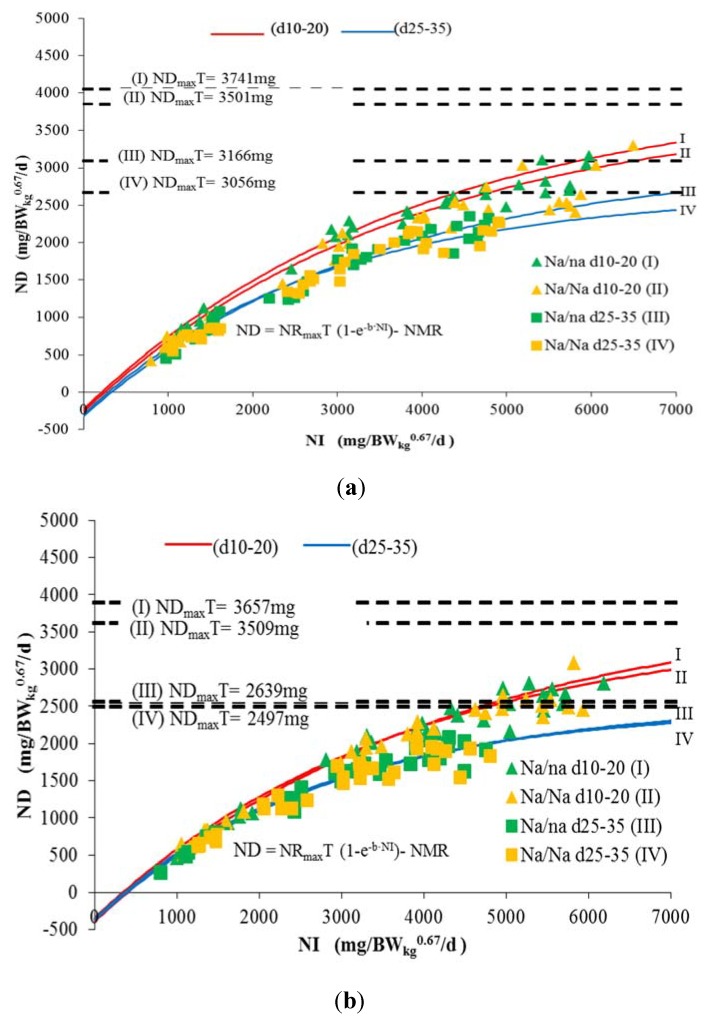
Estimation of the theoretical potential for daily N deposition (ND_max_T) in naked neck chickens of different sexes, genotypes and age periods based on daily N deposition (balance) dependent on daily N intake (NI). NR_max_T = theoretical maximum for daily N retention; e = basic number of natural logarithm (ln); *b* = slope of the N-retention curve (indicating the feed protein quality independent of NI); NMR = N maintenance requirement. (**a**) Males; (**b**) Females.

The threshold values for ND in male and female naked neck growing chickens are demonstrated in Figure 2 and summarized in Table 6. A significant difference was only observed between age periods.

This observed age dependent decline of ND_max_T is in general agreement with earlier observations [13]. However, results reported from modern broiler genotypes [11] indicate disappearance of this decline of ND_max_T with increasing age of the birds. In addition, a trend was observed that the heterozygous chicken of both sexes demonstrated a slightly higher threshold value for the theoretical maximum of ND (ND_max_T). This observation should not be over-interpreted from current point of view.

## 4. Conclusions

In comparison to commercial breeds of meat type chicken, the naked neck chickens should be better adapted to hot climate conditions. Thus may be advantageous [4] to introduce the naked neck gene into fast growing breeds of meat type chickens. If so, the naked neck genotype could be advantageous in developing breeds suitable for environmental conditions with heat stress. However, in addition to improved adaptation to hot climate both acceptable growth performance and efficiency of nutrient utilization are needed. The current study observed that the estimated parameter of theoretical N deposition potential (ND_max_T) of naked neck chickens is slightly below the potential recent commercial genotypes [11] but superior to earlier commercial meat type chickens [13]. From this point of view, meat type chickens of the genotype under study hold an acceptable zoo-technical potential. Accordingly, not acceptable zoo-technical potential of the naked neck genotype are to be expected but acceptable production potential use for efficient broiler production systems. In addition, the current study yielded the first experimental data providing basic parameters (NMR, NR_max_T), which are the precondition for further application of nutritional modeling approaches [21] to derive individual amino acid requirements for the naked neck genotype. Ongoing studies making use of this genotype will help to answer the important question of whether the naked neck gene could modify absolute requirement data for the sulfur containing amino acids methionine and cysteine or the optimal ratio between these amino acids.
